# Manganese-Induced Toxicity in *C. elegans*: What Can We Learn from the Transcriptome?

**DOI:** 10.3390/ijms231810748

**Published:** 2022-09-15

**Authors:** Merle M. Nicolai, Marcello Pirritano, Gilles Gasparoni, Michael Aschner, Martin Simon, Julia Bornhorst

**Affiliations:** 1 Food Chemistry, Faculty of Mathematics and Natural Sciences, University of Wuppertal, Gaußstraße 20, 42119 Wuppertal, Germany; 2 TraceAge–DFG Research Unit on Interactions of Essential Trace Elements in Healthy and Diseased Elderly (FOR 2558), Berlin-Potsdam-Jena-Wuppertal, 14558 Nuthetal, Germany; 3 Molecular Cell Biology and Microbiology, Faculty of Mathematics and Natural Sciences, University of Wuppertal, Gaußstraße 20, 42119 Wuppertal, Germany; 4 Department for Genetics/Epigenetics, Centre for Human and Molecular Biology, Saarland University, Campus Geb. A2 4, 66123 Saarbrücken, Germany; 5 Department of Molecular Pharmacology, Neuroscience, and Pediatrics, Albert Einstein College of Medicine, 1300 Morris Park Avenue, Bronx, NY 10461, USA

**Keywords:** transcriptome analysis, manganese, *Caenorhabditis elegans*, metal response, oxidative stress, stress response

## Abstract

Manganese (Mn) is an essential ubiquitous transition metal and, when occupationally or environmentally overexposed, a well-known risk factor for several neurological pathologies. However, the molecular mechanisms underlying Mn-induced neurotoxicity are largely unknown. In this study, addressing RNA-Seq analysis, bioavailability and survival assays, key pathways of transcriptional responses to Mn overexposure were investigated in the model organism *Caenorhabditis elegans* (*C. elegans*), providing insights into the Mn-induced cellular stress and damage response. Comparative transcriptome analyses identified a large number of differentially expressed genes (DEGs) in nematodes exposed to MnCl_2_, and functional annotation suggested oxidative nucleotide damage, unfolded protein response and innate immunity as major damage response pathways. Additionally, a time-dependent increase in the transcriptional response after MnCl_2_ exposure was identified by means of increased numbers of DEGs, indicating a time-dependent response and activation of the stress responses in Mn neurotoxicity. The data provided here represent a powerful transcriptomic resource in the field of Mn toxicity, and therefore, this study provides a useful basis for further planning of targeted mechanistic studies of Mn-induced neurotoxicity that are urgently needed in the face of increasing industrially caused environmental pollution with Mn.

## 1. Introduction

Over the last years, the industrial use of manganese (Mn) has constantly expanded, causing an increase in the ubiquitous existence of the transition metal in the immediate vicinity of the general population. This, in turn, gives rise to an increasing research interest, as Mn overexposure is associated with various forms of neurodegeneration [1]. At physiological concentrations, Mn is an essential trace element, with Mn(II) and Mn(III) being the most relevant oxidation states in biological systems. The transition metal occurs naturally in the earth’s crust and usually exists as silicates, carbonates and oxides. Aside from an occupational setting, human exposure to the metal is mainly via food or drinking water [2,3]. The adequate intake of Mn is ~3 mg/day for adults, which is easily met due to the rich occurrence of this trace element in vegetables, cereal products and drinking water, whereby regional factors contribute to large differences in local Mn occurrence [4]. Mn is an essential trace element and an important component of several enzyme systems [5,6]. The continuous rise in industrial use of Mn has led to increased release of the metal into the environment, causing short- and long-term health and environmental risks [7]. The acute toxicity of inhaled Mn, most likely occurring in an occupational setting (e.g., mining, Mn alloying, dry battery production, Mn salt production, farming with Mn-containing agrochemicals), can induce acute toxicity of the respiratory tract [2]. The same industrial processes can also lead to increased accumulation of Mn in soil and water, putting both the general population and the environment at risk [2,8]. Countries with the highest Mn deposits include South Africa, Brazil, Russia, Gabon and Australia [7]. Additionally, Mn can be found at high levels in parenteral nutrition [9,10] and infant formula [11,12], putting already vulnerable populations at high risk of Mn overexposure. Various epidemiological studies have shown an association between environmental exposure to Mn and adverse neurological effects in adults and children, which can manifest in impaired movement, cognition and emotional and behavioral responses [2,8]. Many targeted mechanistic studies in rodents, model organisms and cellular systems have been focused on the underlying pathways of Mn-induced (neuro)toxicity, investigating hypotheses that focus on oxidative stress and include many areas—from loss of DNA integrity, lipid peroxidation, protein carbonylation, to decay of dopaminergic neurons (exemplarily, Refs [13,14,15,16,17]). While these studies contribute to the mechanistic understanding of Mn-induced neurodegeneration, further biological pathways affected by this environmental toxicant have yet to be fully characterized.

Due to this gap in the knowledge of basic principles of Mn exposure and toxicity, this study aims to characterize the systemic uptake and transcriptional response to Mn overexposure to not only provide an insight into Mn-induced cellular stress and damage but also possible adaptation mechanisms.

*Caenorhabditis elegans* (*C. elegans*) is a well-established model system used for aging, developmental biology, neurobiology and genetic toxicology research. Using this multicellular soil-dwelling nematode allowed research within the framework of the 3R principles (reduction, replacement and refinement of animal experiments) while still providing a high relevance of biological pathways. Around 60–80% of *C. elegans* genes have homologs in humans [18]. Similar to higher organisms, Mn homeostasis is well regulated in the nematode by several conserved Mn transporters [19,20]. Additionally, the fully sequenced genome, short life cycle and high reproduction rate allow time- and cost-efficient investigations [21,22,23]. For this study, wild-type *C. elegans* were exposed to manganese for different time periods in order to identify the time-dependent response and activation of stress response pathways in Mn neurotoxicity. Furthermore, the data represent a powerful transcriptomic resource for subsequent deeper molecular analysis of the identified DEGs and pathways.

## 2. Results and Discussion

### 2.1. Time-Dependent Uptake and Toxicity of Mn

**Figure 1:**
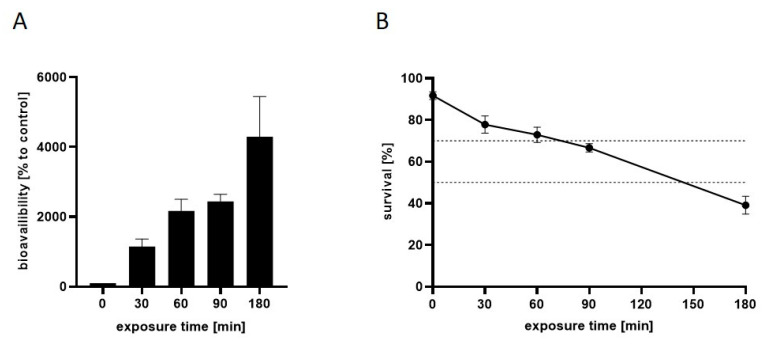
(**A**) Mn bioavailability and (**B**) survival curve of N2 (WT) *C. elegans* after 100 mM MnCl_2_ for up to 180 min. Results show a time-dependent Mn accumulation [% to control] in wild-type nematodes that inversely correlates with the survival rates of the worms analyzed 24 h post-exposure. Data are shown as means ± SEM of four independent experiments.

To assess the time-dependent effects of Mn on the uptake and lethality at overexposure to the transition metal, L4 wild-type *C. elegans* were incubated with 100 mM MnCl_2_ for up to 3 h, and both the bioavailability and the survival rate were analyzed for specific incubation durations (0 min, 30 min, 60 min, 90 min and 180 min). This incubation scheme was based on previous studies that have already confirmed that 60 min Mn exposure at 100 mM is subtoxic, with LD_50_ corresponding to ~200 mM at 60 min exposure [14]. The results presented in Figure 1 indicate a time-dependent uptake of the transition metal [A], which inversely correlates with the survival rate of the nematodes (Pearson correlation, R^2^ = 0.97) [B]. Exposure to 100 mM MnCl_2_ for 60 min caused an increase in the worm Mn levels to over 2000% compared to controls, which, in turn, resulted in a survival rate of 73% (concentration of 100 mM MnCl_2_ for 60 min is shown in Nicolai et al. [14]). This exposure scheme, therefore, was proven optimal for subsequent transcriptome analysis, as it could still be categorized as subtoxic, allowing mechanistic investigations and comparisons of gene expressions at both shorter and longer incubation scenarios. Lethal doses of 30% (LD_30_) and 50% (LD_50_) were reached upon incubations with 100 mM MnCl_2_ for 75 min and 145 min, respectively.

### 2.2. Time Series Transcriptomics during Mn Exposure

**Figure 2:**
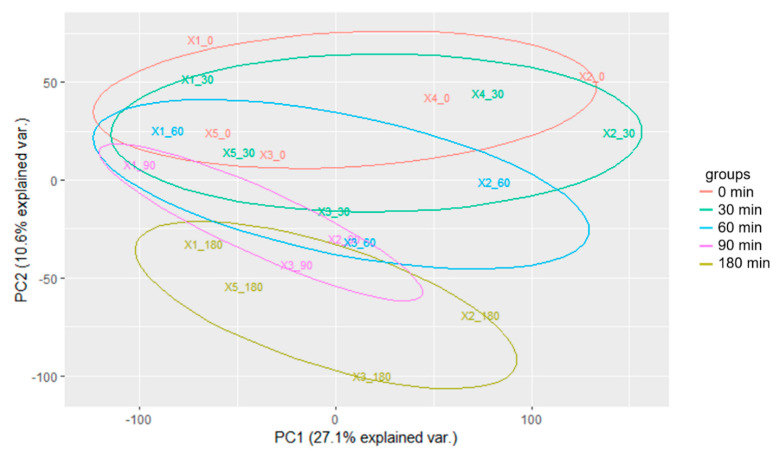
PCA plot demonstrating the increasing divergence of the transcriptome relative to the start of the experiment with increasing exposure time (PC2). Transcriptomic data generated from worms were used, which were exposed to a subtoxic dose of 100 mM MnCl_2_ for 0, 30, 60, 90 and 180 min. Samples in the PCA plot are indicated by the number of the replicate followed by the exposure time to MnCl_2_. Additionally, samples are color coded to highlight replicates of the same exposure time according to the color legend.

To characterize the transcriptional response of worms to Mn, we decided to expose them to the subtoxic dose of 100 mM MnCl_2_. Aiming to discriminate between cellular responses dependent upon the parameters of exposure duration and the cellular uptake, samples for RNA-seq analysis were collected after an Mn exposure time of 30, 60, 90 and 180 min, including a control without Mn exposure (0 min). Each time point is represented by at least three replicates involving 3000 individuals each. After poly-A library preparation, sequencing and quality checks, reads were mapped to the *C. elegans* genome assembly. Differential gene expression analysis was conducted using DESeq2 [24], involving an internal correction of absolute read counts with regard to the library size between the samples [25]. Dimensionality reduction was carried out to gain the first insight into the transcriptomic differences between the samples. Figure 2 shows the principal component analysis (PCA), demonstrating an increased divergence of the transcriptome relative to the start of the experiment with increasing exposure time (PC2). We therefore conclude that the cellular response to Mn exposure is reflected by an ongoing adaption of cellular pathways.

### 2.3. Identification of Differentially Expressed Genes (DEGs) and GO Annotation/Grouping

**Figure 3:**
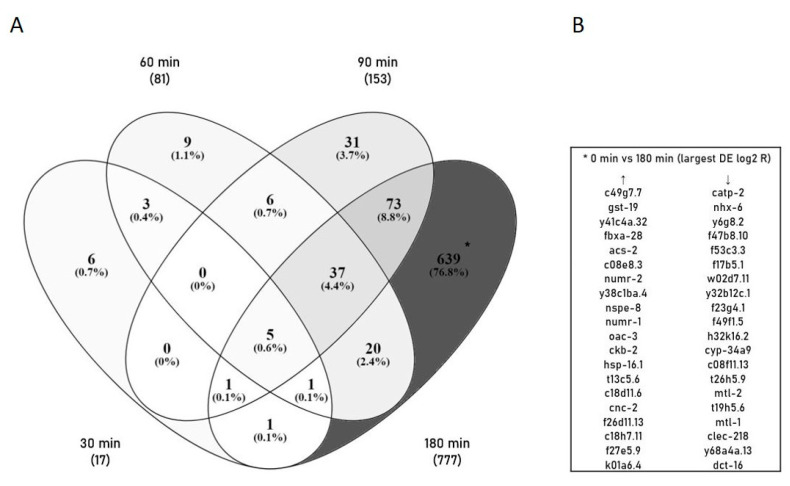
(**A**) Overview of time-specific DEGs at 30 min, 60 min, 90 min and 180 min 100 mM MnCl_2_ exposed *C. elegans* compared to non-exposed controls. Venn diagram identified increase in total number of DEGs with increasing exposure times (30 min vs. 0 min: 17, 60 min vs. 0 min: 81, 90 min vs. 0 min: 153, and 180 min vs. 0 min: 777). (**B**) Additionally, most intense DEGs only present at 180 min vs. 0 min are listed. DEGs were illustrated using *Venny* [25].

To understand the onset of (stress) response and defense mechanisms after excessive Mn exposure, we investigated the time-dependent changes in gene expression. As the Mn uptake and lethality rate in wild-type *C. elegans* were demonstrated to increase with longer exposure times, we wanted to investigate the changes in gene expression over the course of non-toxic to toxic exposure scenarios. Using DESeq2, genes displaying significant changes in gene expression after 30 min, 60 min, 90 min and 180 min of Mn overexposure (100 mM MnCl_2_) were detected. Over 800 differentially expressed genes (DEGs) were identified by comparing the control library (0 min) to Mn-treated time-point libraries. Further, the results indicate a distinct increase in the number of DEGs with increasing incubation time (Figure 3). While only 17 DEGs were identified after a 30 min Mn exposure, the number rises to 81 and 153 at 60 min and 90 min exposure scenarios, respectively. The majority of DEGs (777) occur at 180 min, indicating a stronger gene regulation at long and therefore toxic Mn incubations compared to shorter, subtoxic exposure scenarios. Furthermore, the Venn diagram shows that only five genes were significantly differentially expressed throughout the entire time course compared to non-exposed samples. Instead, the time-specific DEGs might assist in the identification of the stress response at different time points. A list of the 20 most up- or down-regulated DEGs within the largest group (0 min vs. 180 min) is shown in Figure 3B. This includes genes involved in metal binding/transport (metallothioneins, ATPase activity), oxidative stress response (glutathione s-transferase activity), unfolded protein response (UPR, heat shock proteins, IRE1-mediated UPR) and immune response (innate immune response). A complete list of all DEGs at the specific time points/Venn analysis groups can be found in the supplementary information or upon request.

**Figure 4:**
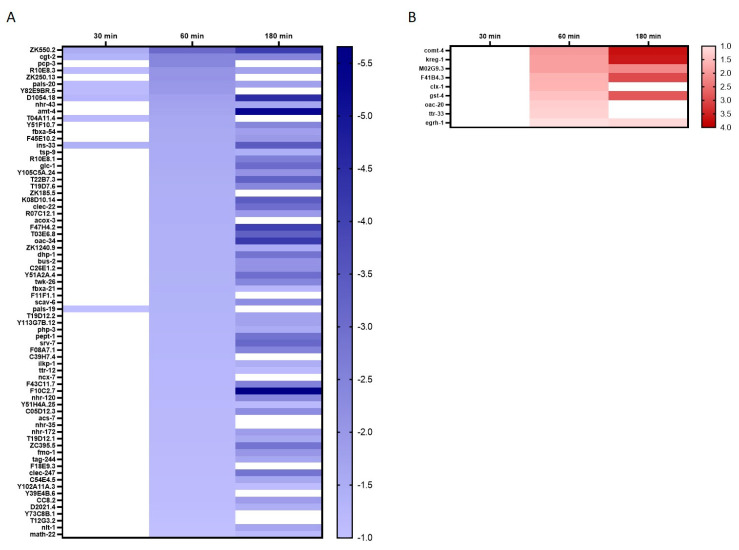
Overview of all DEGs in 0 min vs. 60 min MnCl_2_ exposed wild-type *C. elegans* and their fold changes at 30 min and 180 min compared to non-exposed worms. (**A**) Increased and (**B**) decreased fold changes, analyzed using DESq2. Data are shown as differential expression log2 ratios.

For investigations of fold change during the time course of Mn exposure, we decided to focus on the studies of DEGs present after a 60 min 100 mM MnCl_2_ incubation and compare their fold change with an earlier time point (30 min) and longer incubation (180 min). The 60 min exposure time point was likewise chosen for previous targeted mechanistic studies and allowed gene expression investigations at subtoxic concentrations [14,26]. Figure 4 illustrates the course of fold changes of all DEGs at 60 min 100 mM MnCl_2_ vs. 0 min 100 mM MnCl_2_ and their fold change at 30 min and 180 min. For clarity, genes are sorted as reduced (A) and induced (B) expression compared to control (differential expression log2 ratio), and blank spaces indicate that the respective gene is not significantly different compared to the control at that time point. The majority of DEGs present at 60 min Mn exposure are within the downregulated group, indicating a general beginning of cessation of molecular mechanisms. In both cases of regulation, the fold changes intensify with prolonged incubation time, and most effects are not yet visible after 30 min of MnCl_2_ exposure. Mn-induced oxidative stress, either induced via Fenton-like reactions or by disturbance and impairment of the respiratory chain in mitochondria [27,28], is a widely accepted hypothesis for Mn-induced toxicity. Corroborating previous studies, we were able to show that also in *C. elegans*, incubation with 100 mM MnCl_2_ for 60 min caused significant changes in the glutathione status, energy-related nucleotide ratios and oxidative DNA damage occurrence, indicating a significant increase in reactive oxygen and nitrogen species (RONS) in the nematodes after excessive Mn exposure [14,26]. This was also confirmed by the DEG analysis shown here. Genes for both the antioxidant system (*gst-4*, glutathione s-transferase) and the UPR (*F41V4.3*, upstream of the IRE1-mediated UPR) were significantly increased after 60 min and 180 min Mn exposure. Mn has been extensively associated with movement and cognitive impairment, generally referred to as manganism. In human and rodent studies, it has been shown that Mn accumulates mainly in dopamine-rich areas of the brain, such as the *substantia nigra*, and Mn overexposure is associated with dopaminergic neurodegeneration and decreased abundance of the neurotransmitter, dopamine [13,29,30], similar to those in Parkinson´s disease. Therefore, the relevance of Parkinson’s-disease-associated genes in Mn toxicity was addressed in *C. elegans* previously [31]. While we could not identify any direct links of the DEGs at 60 min MnCl_2_ exposure in these *C. elegans* samples with Parkinson’s-disease-associated genes, the results indicate an increase in the homolog for human EGR1 gene (*egrh-1*, early growth factor response factor 1) implicated in neurological disorders, such as Alzheimer´s disease and regulation of the acetylcholinergic system. EGR1 is able to bind to the promoter region of choline-acetyl transferase, increase its expression and therefore induce the acetylcholine formation [32]. At the same time, EGR1 is implicated in promoting amyloid-β peptide synthesis by transcription activation of the β-secretase 1 (BACE-1) [33], responsible for cleaving the amyloid precursor protein and therefore driving amyloid-β synthesis [34]. The progressive accumulation of amyloid-β in the brain is strongly implicated in the pathogenesis of Alzheimer´s disease [35], and while a few studies have already begun to investigate the link between Mn, acetylcholine, Alzheimer’s disease and amyloid-β [36,37], further research is strongly required.

### 2.4. GO Annotation Identified Oxidative RNA Damage, Unfolded Protein Response and Innate Immunity As Major Pathways in Mn Toxicity

**Figure 5:**
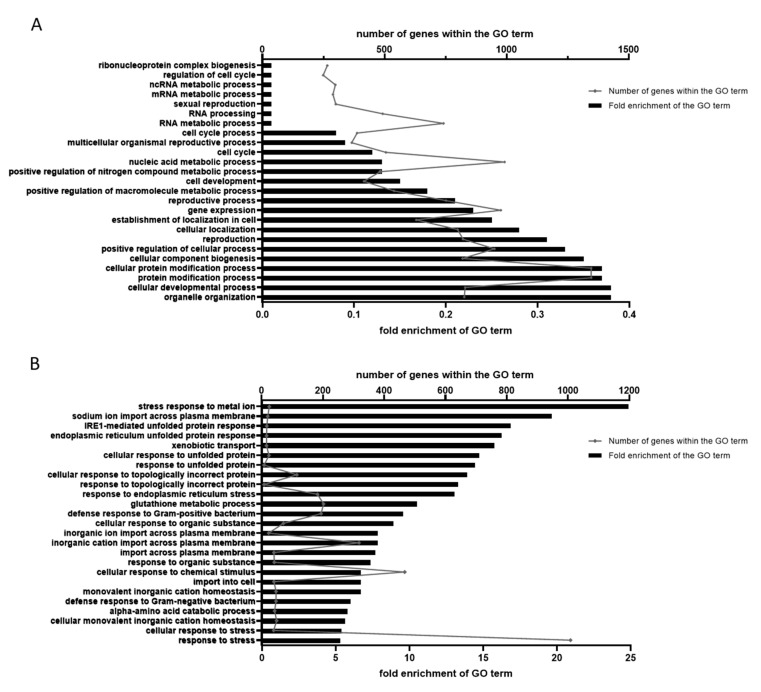
GO annotation of DEGs identified at 180 min 100 mM MnCl_2_ vs. 0 min 100 mM MnCl_2_. (**A**) GO terms of genes with decreased and (**B**) increased expression levels in the 180 min samples compared to the control. Fold enrichment shows to what extent the number of detected genes of a specific GO term differentiated from the expected number of genes.

As most DEGs are within the group of 0 min 100 mM MnCl_2_ vs. 180 min 100 mM MnCl_2_ (DEGs_180_), these genes were used for GO functional annotation, allowing for a better understanding of the biological function and gene interactions. All DEGs_180_ were annotated using geneontology.org into GO terms of biological processes before conducting enrichment analysis. More than 120 GO terms were identified, whereby most annotation groups indicated a decrease in enrichment compared to non-exposed controls. The 20 strongest negatively and positive enriched pathways are shown in Figure 5 and expand the results described above. The GO terms of downregulated genes (Figure 5A) indicate a general diminution of cell-cycle processes, cell differentiation, cellular and organelle development and reproduction (e.g., RNA processing, mRNA metabolic processes and cell-cycle processes/regulation). A strong upregulation (Figure 5B) of the processes involved in ER stress and the antioxidant response further support the hypothesis of Mn-induced oxidative stress. Interestingly, the pathways of the (innate) immune response were likewise upregulated, which could be linked to oxidative DNA damage [38] and provides another interesting pathway for further studies.

Increased levels of RONS caused by the state of oxidative stress are known to cause increased interactions with various macromolecules. While lipids, proteins and DNA have all been investigated as endpoints of Mn toxicity, and adverse oxidative modifications have been detected not only in *C. elegans* but also in cell culture systems, rodents and humans [14,15,39,40] the oxidative modifications of the RNA processing system and the RNA itself are often overlooked. In fact, in situ immunohistochemistry studies identified oxidized nucleosides in cytoplasmic RNA, and not mitochondrial or nuclear DNA, as the predominant oxidative damage in neurodegeneration, such as Alzheimer´s and Parkinson´s diseases [41,42,43]. Kong et al. even observed highly specific mRNA oxidation, independent of their abundance [44]. The GO analyses presented here are in line with adverse effects on translational processes (reduced enrichment in RNA processing, mRNA metabolism and ncRNA metabolism) by oxidative mRNA ribosome stalling and slowing down of translation, thereby causing reduced protein expression [45,46]. Additionally, alterations of mRNA can lead to the production of short polypeptides and defective proteins by translation errors and in combination with RONS-induced protein aggregates, and misfolded proteins might be the cause of the increased fold expression of UPR and endoplasmic reticulum (ER) pathways (IRE1-mediated UPR, ER unfolded protein response, unfolded protein response and response to ER stress) after Mn overexposure [47,48].

Parts of the ER stress response components are also contained within the immune signaling pathways (e.g., NFκB, STATs and JNK), and an upregulation of the innate immunity after Mn exposure, as seen in Figure 5, might be explained by an upregulation of the IRE1-dependent UPR [49]. While *C. elegans* lack some important immune defense systems found in mammals (such as an adaptive immune system or mobile immune cells), and homologs of some key genes, such as NFκB, TLR and vertebrate cytokines, are not conserved in the nematode, other important innate immunity pathways are highly conserved. This includes p38 MAPK, β-catenin and FOXO transcription factors [50,51]. In addition, earlier studies have identified increased oxidative DNA damage and DNA damage response after Mn exposure, which, in turn, has been shown to activate the innate immune response in *C. elegans* [14,15,38].

### 2.5. The Transcriptional Response to Mn Overexposure Increases with Time and Bioavailability

**Figure 6:**
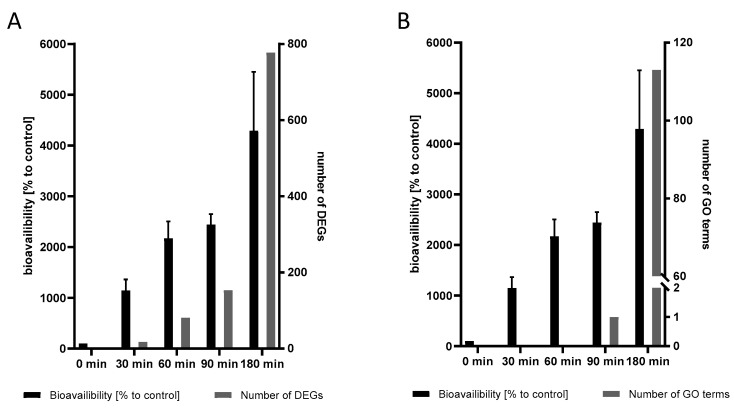
Time course of Mn bioavailability and (**A**) number of DEGs and (**B**) number of GO terms. Bioavailability data are shown as means ± SEM of four independent experiments.

Using the bioavailability assessment of Mn, it was shown that with increasing exposure durations (from 0 min to 180 min), the Mn content of the worms was increased in a time-dependent manner (described in detail above). This was accompanied by a steady increase in the transcriptional damage response, as shown in Figure 6. Both the number of DEGs for each time point (A) and the number of GO terms (B) increased in a non-linear manner with longer incubation times and resulting in higher bioavailability levels. This, in turn, might indicate not only an increasing onset of damage response pathways, but also the downregulation of normal cellular processes.

## 3. Materials and Methods

### 3.1. C. elegans Cultivation, Mn Treatment and Assessment of Toxicity

For the assessment of Mn toxicity and transcriptome analysis, the wild-type (WT) N2 Bristol *C. elegans* strain was used, which was provided by the *Caenorhabditis elegans Center* (CGC; University of Minnesota, Minneapolis, MN, USA).

Synchronized larval stage 4 (L4) worm populations were used for all experimental setups. For the generation of these populations, *C. elegans* were cultivated on agar plates at 20 °C, as described by Brenner (1974) [52]. After synchronization using 20% bleach in dH_2_O (+1.5 M NaOH) and seeding of L1 (larval stage 1) on OP50 *E. coli*-covered NGM plates, the worms were allowed to develop into L4 without further interference.

Before Mn treatment, worms were washed with 85 mM NaCl + 0.1% Tween (Sigma Aldrich, Steinheim, Germany) to remove all bacteria. A total of 3000 N2 (WT) per sample were incubated with 100 mM MnCl_2_ for different exposure times (0 min, 30 min, 60 min, 90 min and 180 min) in liquid (85 mM NaCl) while slowly rotating. In detail, all worms started to rotate in 85 mM NaCl at the start of the experiments. For the 180 min Mn treatment, Mn was added directly to the worms. For the 90 min treatment, Mn was added 90 min after the rotation in 85 mM NaCl started. For the 60 min treatment, Mn was added 120 min after the rotation in 85 mM NaCl started. For the 30 min treatment, Mn was added 150 min after the rotation in 85 mM NaCl started. The non-treated worms rotated for the whole 180 min in order to provide the same conditions in all samples, which allowed us to evaluate Mn-specific effects.

After 180 min, worms were washed three times with 85 mM NaCl + 0.01% Tween for further analysis. MnCl_2_ was purchased from Sigma-Aldrich (Steinheim, Germany) (purity > 99.995%) and diluted in 85 mM NaCl.

Mn toxicity at different exposure lengths was assessed using the lethality assay. For this, the exposed *C. elegans* were transferred to OP50-covered NGM plates, and dead and alive worms were counted manually 24 h post-exposure. The vitality of the worms was checked using the mechanic stimulus of touch using a platinum wire. Worms that did not respond to the stimulus were considered dead.

### 3.2. Measurement of Mn Bioavailability

Mn bioavailability was assessed using inductively coupled plasma–optical emission spectrometry (ICP-OES, (Spectro, Krefeld, Germany)) as described previously [14]. In short, samples were homogenized (3× freeze–thaw cycles and sonication), dried and acid assisted digested with HNO_3_:H_2_O_2_; (1:1) at 95 °C. The analytical instrument settings were chosen as follows: plasma power: 1400 W, refrigerant gas flow: 12 L/min, auxiliary gas flow: 1 L/min, nebulizer gas type and flow: MicroMist, 1 L/min, wavelength: 257.611 nm. An external calibration using a multi-element mix (Spetec-645) and the Spectro Smart Analyzer were used for quantification and analysis. Measurement accuracies were assessed using single-cell protein standards certified for trace elements (Institute for Reference Materials and Measurement of the European Commission, Geel, Belgium). All samples were normalized to protein content measured by BCA analysis (bicinchoninic acid assay kit (Thermo Scientific, Waltham, MA, USA)).

### 3.3. Sample Preparation for RNA Sequencing

Directly after Mn exposure and washing, the 3000 Mn-exposed worms were pelletized, and 1 mL Tri Reagent (Invitrogen, Thermo Fischer Scientific, Waltham, MA, USA) was added to each sample. After homogenization, samples were stored at −80 °C until further sample preparation.

RNA was isolated using the Direct-zol RNA Miniprep kit (Zymo Research; #R2050, Freiburg, Germany). For this, the manufacturer’s instructions were adapted to *C. elegans* samples. In short, 2 freeze–thaw cycles using liquid nitrogen and 37 °C water bath were conducted before mixing the samples well and transferring 500 µL of the sample to the Zymo spin column. Spin columns were centrifuged, the flow-through discarded, and columns were washed with 400 µL RNA wash buffer. DNase I was added to all samples and incubated for 15 min at room temperature. Columns were washed again with RNA (pre-)wash buffer before eluting the RNA in 25 µL DNA/RNAse free water. RNA concentrations were measured using the Invitrogen^™^ Qubit^™^ system (Thermo Fisher, Waltham, MA, USA), and RNA integrity was verified using Agilent Bioanalyzer RNA pico chips.

### 3.4. Transcriptome Analysis

Library preparation was performed using the CATS mRNA-seq Kit (with polyA selection) v2 (Diagenode Cat# C05010043, Leuven, Belgium) according to the manufacturer’s instructions. Libraries were pooled and single end sequenced on the HiSeq2500 Illumina platform in high output mode with 96 cycles. Reads were demultiplexed, and the template switch motif as well as poly-A-tails and adapter sequences were removed using Cutadapt V2.10 [53]. Raw reads are available at the European Nucleotide Archive (ENA) under accession nr. PRJEB52734 (ERP137472).

Reads were analyzed using the Geneious Prime^®^ Software V.2020.2.4 (by Dotmatics/Biomatters, Ltd., Auckland, New Zealand, d Mapping was performed using the Bowtie2 [54] plugin (plugin Version 7.2.1) in End-to-End mode using the Bristol N2 genome sequence as a template. Transcription levels were calculated using the Geneious Prime^®^ V.2020.2.4 “calculate expression levels” tool.

### 3.5. DEG Analysis of Time Course Samples

To visualize the general differences between the samples after varying periods of MnCl_2_ exposure, principal component analysis was performed using the TPM values of all annotated protein-coding genes using R, visualized with the ggbiplot library.

Differentially expressed genes after MnCl_2_ exposure were calculated using the DESeq2 [24] plugin of the Geneious Prime^®^ Software V.2020.2.4, comparing each individual exposure time to the reference samples without MnCl_2_ exposure (indicated as time: 0 min; adjusted *p*-value cut-off: 0.01; minimal fold change: 2). Using the aforementioned DEGs, the enriched GO terms for up- and down-regulated genes after exposure were determined by the PANTHER web tool ([55,56] Available online: http://geneontology.org/; accessed on 14 March 2021). The GO terms were visualized using GraphPad Prism 9. The Venn diagram showing the number of DEGs at the different time points was created using *Venny* [25].

For DEGs that changed their expression after 60 min of MnCl_2_ exposure, a heat map was used to show the change in expression levels between time points 0 min, 60 min and 180 min to look for genes with similar expression change patterns. The heat map was created by using GraphPad Prism 9.

## 4. Conclusions

Chronic environmental exposure to high levels of Mn is a well-established risk factor for the etiology of severe, atypical parkinsonian syndrome (manganism) with underlying mechanisms that are still debated and not understood. While most studies are based on targeted hypotheses to address the mechanism of Mn toxicity, a comparative transcriptome analysis allows identifying the candidate genes involved in Mn toxicity. The transcriptome analysis of the model *C. elegans* exposed toward Mn clearly indicates the relevance of specific pathways involved in Mn toxicity, such as oxidative stress, the antioxidant response, ER stress and immune signaling pathways. Additionally, analyzing the transcriptional response and Mn content in the worm over various durations of exposure allows the identification of pathways and counter-regulating mechanisms of toxicity (possible detoxification pathways). The transcriptome analysis described here will help in the field of Mn-induced neurotoxicity to plan and build on specific targeted experimental set-ups needed for identification of the underlying mechanisms of Mn neurotoxicity.

## Supplementary Materials

The following are available online at https://www.mdpi.com/article/10.3390/ijms231810748/s1.

## References

[1] O’Neal S.L., Zheng W. (2015). Manganese Toxicity Upon Overexposure: A Decade in Review. Curr. Environ. Health Rep..

[2] Williams M., Todd G.D., Roney N., Crawford J., Coles C., McClure P.R., Garey J.D., Zaccaria K., Citra M. (2012). Agency for Toxic Substances and Disease Registry (ATSDR) Toxicological Profiles. Toxicological Profile for Manganese.

[3] SCoF E. (2006). Tolerable Upper Intake Levels for Vitamins and Minerals. Scientific Panel on Dietetic Products, Nutrition and Allergies.

[4] European Food Safety Authority (2013). Scientific Opinion on Dietary Reference Values for manganese. EFSA J..

[5] Chen P., Bornhorst J., Aschner M. (2018). Manganese metabolism in humans. Front. Biosci..

[6] Mattison D.R., Milton B., Krewski D., Levy L., Dorman D.C., Aggett P.J., Roels H.A., Andersen M.E., Karyakina N.A., Shilnikova N. (2017). Severity scoring of manganese health effects for categorical regression. Neurotoxicology.

[7] Rollin H.B., Nogueira C.M.C.A. (2011). Manganese: Environmental Pollution and Health Effects 2011.

[8] Howe P.D., Malcolm H.M., Dobson S., World Health O., International Programme on Chemical S. (2004). Manganese and Its Compounds: Environmental Aspects.

[9] Fitzgerald K., Mikalunas V., Rubin H., McCarthey R., Vanagunas A., Craig R.M. (1999). Hypermanganesemia in patients receiving total parenteral nutrition. JPEN J. Parenter. Enteral. Nutr..

[10] Abdalian R., Saqui O., Fernandes G., Allard J.P. (2013). Effects of manganese from a commercial multi-trace element supplement in a population sample of Canadian patients on long-term parenteral nutrition. JPEN J. Parenter. Enteral. Nutr..

[11] Scher D.P., Goeden H.M., Klos K.S. (2021). Potential for Manganese-Induced Neurologic Harm to Formula-Fed Infants: A Risk Assessment of Total Oral Exposure. Environ. Health Perspect..

[12] Aschner J.L., Aschner M. (2005). Nutritional aspects of manganese homeostasis. Mol. Asp. Med..

[13] Lin M., Colon-Perez L.M., Sambo D.O., Miller D.R., Lebowitz J.J., Jimenez-Rondan F., Cousins R.J., Horenstein N., Aydemir T.B., Febo M. (2020). Mechanism of Manganese Dysregulation of Dopamine Neuronal Activity. J. Neurosci..

[14] Nicolai M.M., Weishaupt A.K., Baesler J., Brinkmann V., Wellenberg A., Winkelbeiner N., Gremme A., Aschner M., Fritz G., Schwerdtle T. (2021). Effects of Manganese on Genomic Integrity in the Multicellular Model Organism Caenorhabditis elegans. Int. J. Mol. Sci..

[15] Nicolai M.M., Witt B., Friese S., Michaelis V., Hölz-Armstrong L., Martin M., Ebert F., Schwerdtle T., Bornhorst J. (2022). Mechanistic studies on the adverse effects of manganese overexposure in differentiated LUHMES cells. Food Chem. Toxicol..

[16] Erikson K.M., Dobson A.W., Dorman D.C., Aschner M. (2004). Manganese exposure and induced oxidative stress in the rat brain. Sci. Total Environ..

[17] Rudgalvyte M., Peltonen J., Lakso M., Nass R., Wong G. (2016). RNA-Seq Reveals Acute Manganese Exposure Increases Endoplasmic Reticulum Related and Lipocalin mRNAs in Caenorhabditis elegans. J. Biochem. Mol. Toxicol..

[18] Kaletta T., Hengartner M.O. (2006). Finding function in novel targets: *C. elegans* as a model organism. Nat. Rev. Drug Discov..

[19] Au C., Benedetto A., Anderson J., Labrousse A., Erikson K., Ewbank J.J., Aschner M. (2009). SMF-1, SMF-2 and SMF-3 DMT1 orthologues regulate and are regulated differentially by manganese levels in *C. elegans*. PLoS ONE.

[20] Chakraborty S., Chen P., Bornhorst J., Schwerdtle T., Schumacher F., Kleuser B., Bowman A.B., Aschner M. (2015). Loss of pdr-1/parkin influences Mn homeostasis through altered ferroportin expression in *C. elegans*. Metallomics.

[21] Corsi A.K., Wightman B., Chalfie M. (2015). A Transparent Window into Biology: A Primer on Caenorhabditis elegans. Genetics.

[22] Lai C.H., Chou C.Y., Ch’ang L.Y., Liu C.S., Lin W. (2000). Identification of novel human genes evolutionarily conserved in Caenorhabditis elegans by comparative proteomics. Genome Res..

[23] Hunt P.R. (2017). The *C. elegans* model in toxicity testing. J. Appl. Toxicol..

[24] Love M.I., Huber W., Anders S. (2014). Moderated estimation of fold change and dispersion for RNA-seq data with DESeq2. Genome Biol..

[25] Oliveros J.C. (2007–2015). Venny. An Interactive Tool for Comparing Lists with Venn’s Diagrams. https://bioinfogp.cnb.csic.es/tools/venny/index.html.

[26] Neumann C., Baesler J., Steffen G., Nicolai M.M., Zubel T., Aschner M., Burkle A., Mangerich A., Schwerdtle T., Bornhorst J. (2020). The role of poly(ADP-ribose) polymerases in manganese exposed Caenorhabditis elegans. J. Trace Elem. Med. Biol..

[27] Malecki E.A. (2001). Manganese toxicity is associated with mitochondrial dysfunction and DNA fragmentation in rat primary striatal neurons. Brain Res. Bull..

[28] Galvani P., Fumagalli P., Santagostino A. (1995). Vulnerability of mitochondrial complex I in PC12 cells exposed to manganese. Eur. J. Pharmacol..

[29] Morello M., Canini A., Mattioli P., Sorge R.P., Alimonti A., Bocca B., Forte G., Martorana A., Bernardi G., Sancesario G. (2008). Sub-cellular localization of manganese in the basal ganglia of normal and manganese-treated rats An electron spectroscopy imaging and electron energy-loss spectroscopy study. Neurotoxicology.

[30] Balachandran R.C., Mukhopadhyay S., McBride D., Veevers J., Harrison F.E., Aschner M., Haynes E.N., Bowman A.B. (2020). Brain manganese and the balance between essential roles and neurotoxicity. J. Biol. Chem..

[31] Bornhorst J., Chakraborty S., Meyer S., Lohren H., Große Brinkhaus S., Knight A.L., Caldwell K.A., Caldwell G.A., Karst U., Schwerdtle T. (2013). The effects of pdr1, djr1.1 and pink1 loss in manganese-induced toxicity and the role of α-synuclein in *C. elegans*. Metallomics.

[32] Quirin-Stricker C., Mauvais C., Schmitt M. (1997). Transcriptional activation of human choline acetyltransferase by AP2- and NGF-induced factors. Brain Res. Mol. Brain Res..

[33] Qin X., Wang Y., Paudel H.K. (2016). Early Growth Response 1 (Egr-1) Is a Transcriptional Activator of β-Secretase 1 (BACE-1) in the Brain. J. Biol. Chem..

[34] Vassar R., Kovacs D.M., Yan R., Wong P.C. (2009). The beta-secretase enzyme BACE in health and Alzheimer’s disease: Regulation, cell biology, function, and therapeutic potential. J. Neurosci. Off. J. Soc. Neurosci..

[35] Hampel H., Hardy J., Blennow K., Chen C., Perry G., Kim S.H., Villemagne V.L., Aisen P., Vendruscolo M., Iwatsubo T. (2021). The Amyloid-β Pathway in Alzheimer’s Disease. Mol. Psychiatry.

[36] Lin G., Li X., Cheng X., Zhao N., Zheng W. (2020). Manganese Exposure Aggravates β-Amyloid Pathology by Microglial Activation. Front. Aging Neurosci..

[37] Tong Y., Yang H., Tian X., Wang H., Zhou T., Zhang S., Yu J., Zhang T., Fan D., Guo X. (2014). High manganese, a risk for Alzheimer’s disease: High manganese induces amyloid-β related cognitive impairment. J. Alzheimers Dis..

[38] Nakad R., Schumacher B. (2016). DNA Damage Response and Immune Defense: Links and Mechanisms. Front. Genet..

[39] Yiin S.-J., Lin T.-H., Shih T.-S. (1996). Lipid peroxidation in workers exposed to manganese. Scand. J. Work. Environ. Health.

[40] Cai T., Yao T., Li Y., Chen Y., Du K., Chen J., Luo W. (2007). Proteasome inhibition is associated with manganese-induced oxidative injury in PC12 cells. Brain Res..

[41] Nunomura A., Perry G., Pappolla M.A., Wade R., Hirai K., Chiba S., Smith M.A. (1999). RNA oxidation is a prominent feature of vulnerable neurons in Alzheimer’s disease. J. Neurosci. Off. J. Soc. Neurosci..

[42] Chang Y., Kong Q., Shan X., Tian G., Ilieva H., Cleveland D.W., Rothstein J.D., Borchelt D.R., Wong P.C., Lin C.-L.G. (2008). Messenger RNA oxidation occurs early in disease pathogenesis and promotes motor neuron degeneration in ALS. PLoS ONE.

[43] Nunomura A., Chiba S., Kosaka K., Takeda A., Castellani R.J., Smith M.A., Perry G. (2002). Neuronal RNA oxidation is a prominent feature of dementia with Lewy bodies. Neuroreport.

[44] Kong Q., Lin C.-L.G. (2010). Oxidative damage to RNA: Mechanisms, consequences, and diseases. Cell Mol. Life Sci..

[45] Ding Q., Dimayuga E., Keller J.N. (2007). Oxidative stress alters neuronal RNA- and protein-synthesis: Implications for neural viability. Free Radic. Res..

[46] Shan X., Chang Y., Lin C.G. (2007). Messenger RNA oxidation is an early event preceding cell death and causes reduced protein expression. FASEB J..

[47] Tanaka M., Chock P.B., Stadtman E.R. (2007). Oxidized messenger RNA induces translation errors. Proc. Natl. Acad. Sci. USA.

[48] Abramov A.Y., Potapova E.V., Dremin V.V., Dunaev A.V. (2020). Interaction of Oxidative Stress and Misfolded Proteins in the Mechanism of Neurodegeneration. Life.

[49] Di Conza G., Ho P.-C. (2020). ER Stress Responses: An Emerging Modulator for Innate Immunity. Cells.

[50] Irazoqui J.E., Urbach J.M., Ausubel F.M. (2010). Evolution of host innate defence: Insights from Caenorhabditis elegans and primitive invertebrates. Nat. Rev. Immunol..

[51] Pukkila-Worley R., Ausubel F.M. (2012). Immune defense mechanisms in the Caenorhabditis elegans intestinal epithelium. Curr. Opin. Immunol..

[52] Brenner S. (1974). The genetics of Caenorhabditis elegans. Genetics.

[53] Martin M. (2011). Cutadapt removes adapter sequences from high-throughput sequencing reads. EMBnet. J..

[54] Langmead B., Salzberg S.L. (2012). Fast gapped-read alignment with Bowtie 2. Nat. Methods.

[55] Mi H., Ebert D., Muruganujan A., Mills C., Albou L.-P., Mushayamaha T., Thomas P.D. (2021). PANTHER version 16: A revised family classification, tree-based classification tool, enhancer regions and extensive API. Nucleic Acids Res..

[56] Thomas P.D., Kejariwal A., Guo N., Mi H., Campbell M.J., Muruganujan A., Lazareva-Ulitsky B. (2006). Applications for protein sequence-function evolution data: mRNA/protein expression analysis and coding SNP scoring tools. Nucleic Acids Res..

